# Compliance with retainer wear using audiovisual integration and reminder: a randomized clinical trial

**DOI:** 10.1038/s41598-023-35686-4

**Published:** 2023-05-26

**Authors:** Khaled Wafaie, Hisham Mohammed, Wang Xinrui, Jinshu Zhou, Ahmed M. El Sergani, Qiao Yiqiang

**Affiliations:** 1 Department of Orthodontics, Faculty of Dentistry, First Affiliated Hospital of Zhengzhou University, No. 1 Jianshe East Road, Erqi District, Zhengzhou, Henan China; 2 Department of Oral Sciences, Faculty of Dentistry, University of Otago, Dunedin, New Zealand; 3 Department of Oral and Craniofacial Sciences, University of Pittsburgh School of Dental Medicine, Pittsburgh, USA

**Keywords:** Dentistry, Disease prevention, Patient education, Quality of life

## Abstract

Active audiovisual representation of instructions ensures vibrant knowledge acquisition and improves acquaintance needed for self-care with retainer wear. The aim of this trial is to assess the impact of audiovisual instructions with additional weekly electronic reminder messages on improving adherence to instructed wear time of Hawley retainer, periodontal outcomes, and participants’ experiences. Fifty-two participants (mean age 26.1 y) planned for removable retention, were randomly assigned to two parallel groups to receive either (1) audiovisual instructions with an additional weekly reminder, or (2) verbal instructions alone. Each participant received a Hawley retainer equipped with a TheraMon microsensor and was instructed to wear it for 22 h daily. Participants were monitored for adherence to the wear time after 3 (T1) and 6 months (T2), and had their periodontal health and experiences assessed at T2. Overall, the mean objectively measured daily wear time at T1 was 14.9 (± 4.9 h), and 14.3 (± 5.4 h) at T2. After 3 months, no significant differences were found between the groups (*p* = 0.065), however, a significant difference favoring better compliance with wear instructions was observed in the audiovisual group after 6 months (*p* = 0.033). A non-significant difference was observed between both groups regarding the gingival (*p* = 0.165) and plaque index scores (*p* = 0.173). Participants’ experiences were similar in both groups, except for satisfaction with the way of delivering instructions, being favorably reported in the audiovisual group. Audiovisual instructions with weekly reminders seem to have a significant effect on patient compliance in the longer term.

**Trial registration:** TCTR20230220002.

## Introduction

Since the nineteenth century to date, relapse has been one of the most difficult and unsolved problems in orthodontics. Some degree of relapse is inevitable and more deleterious in some patients in comparison to others^1^. The amount of relapse varies from 0.6 to 3.5 mm and is expected in almost 70% of patients, according to the duration and type of retention^2^. Consequently, removable, fixed, or even dual retainers are prescribed to orthodontic patients. Further understanding of the complex array of interplay between skeletal, dental, and soft tissue relationships, could guide us to the choice of retention^3^. For removable retainers, patients need to comply with prescribed wearing hours to prevent relapse^4^. Compliance with removable appliances empirically is sub-optimal and is expected in only 8% of patients^5^. Objectively measured wear time by microsensors accounts for 50% of the prescribed wear time^6,7^.

Factors affecting compliance have been previously investigated in the literature^8^. For instance, failure to recall wearing the retainer occurs in almost half of the patients. Both vacuum-formed and Hawley retainers cause discomfort with speech, especially in the first few weeks, until patients adapt to wearing them^9^. The appearance of the appliances causes embarrassment to some patients, especially with the Hawley retainer, which has a metal labial bow^10^. Furthermore, the method of acquisition and retention of knowledge could affect the adherence level^11^. Few studies have assessed the effect of interventional methods on increasing compliance. From these methods, the Hawthorne effect was found to be a convenient way to improve compliance^12,13^. Another reported method is conscious hypnosis, which was used by hypnotherapists to induce the patients based on what they aim to achieve^14^. The use of calendars as reminders has been evaluated in one trial showing a positive effect on compliance^15^. The utilization of visual images instead of verbal instructions alone was assessed in one trial, however, self-reporting of results is subjective and inaccurate^16^.

Accordingly, to achieve better compliance, new behavioral modeling interventions need to be evaluated in orthodontics. For instance, in self-efficacy theory, patients’ expectations about their ability to comply depend on their clear understanding of the desired outcomes^17^. Hence, patients could see appliances/attachments during treatment; feel the pressure or pain, and follow up the progress. However, the retention phase mainly relies on monitoring. Thus, delivering long term clear goals is mandatory to achieve consistent compliance with the retainer. Improving the method of delivering information is vital in the process. Traditional unimodal methods (verbal/written), have been used for many years to deliver the aims of treatment and instructions for self-care. However, their limitations disempower patients’ ability to refer to information over the long term, making them unreliable^18^.

An alternative method is a multimodal approach, which mixes auditory and visual senses (audiovisual) to induce a positive expressive reaction that results in a relaxing behavior and full capacity for concentration^19^. This method could enhance the retention of knowledge in the short term and is superior in terms of learning in comparison to single modalities^20,21^. It also helps to facilitate behavioral modeling which makes adapting to new habits easier^22^. An additional method is the reminder system, which depends on the Hawthorne effect^23^. Reminders have been utilized in a variety of forms, including mobile applications, computer-generated SMS, mobile messages, phone calls, personalized calendar sheets, or even postal letters to reshape patients' behavior^24,25^. Literature has shown positive effects of reminders on modifying oral hygiene parameters over the short and long term, and improving adherence to appointments resulting in an overall shorter orthodontic treatment duration^13,25^. Furthermore, patients were found to have a preference for weekly reminders rather than daily ones, and that weekly reminders were found to have a profound effect^25^. However, they do seem to have a limited impact during retention^26^. Accordingly, a combination of reminders and audiovisual integration could retain information for longer periods.

### Aim

Our primary aim was to assess the impact of audiovisual instructions with additional weekly electronic reminder messages, on improving the adherence to instructed wear time of Hawley retainer, in comparison to verbal instructions alone. The secondary outcomes were to assess gingival and periodontal health after 6 months, and to assess participants’ experiences and effect on behavioral compliance that could henceforth be identified.

## Methods

### Study design

**Figure 1:**
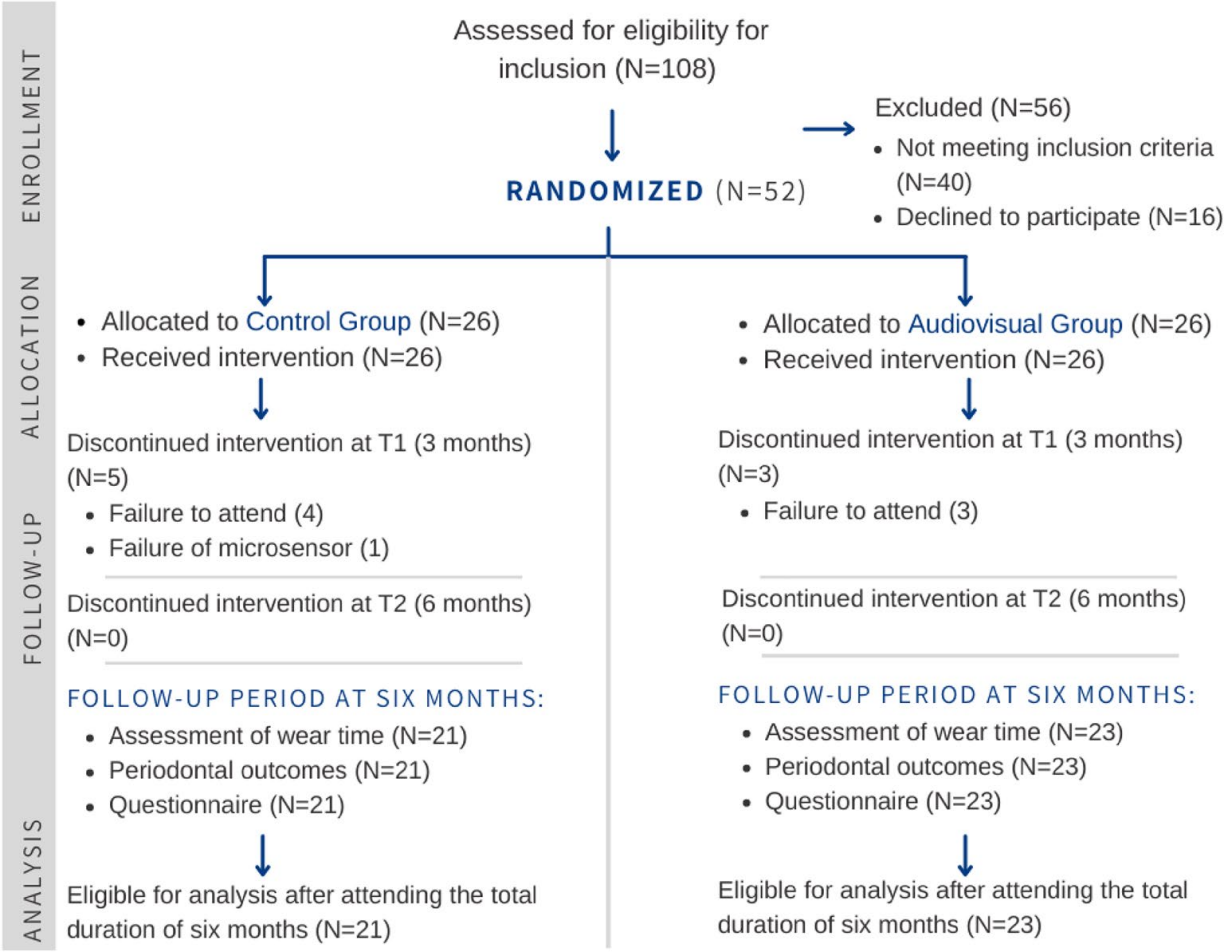
Consort flow diagram.

This was a double-arm parallel-group prospective superiority randomized clinical trial, performed in a single center at the orthodontic department of the First Affiliated Hospital of Zhengzhou University from November 19, 2020, to October 12, 2022. Prior to the study commencement, ethical approval was obtained from the ethical committee at Zhengzhou University with reference number (2021-KY-1026-002). All methods were performed in accordance with the relevant guidelines and regulations of the ethical committee. The trial was registered with the Thai Clinical Trials Registry (TCTR) with trial number (TCTR20230220002). Participants who met the inclusion criteria were recruited prior to debonding. The inclusion criteria were: participants aged 16–35 years, and have a smartphone with the capability to use the WeChat application. The exclusion criteria were: cleft lip or palate; systemic disease or medical condition; periodontal disease; craniofacial deformities; taking any medication that affects gingival health; and history of orthodontic treatment and retention. All participants were informed about the study objectives and signed the respective consent forms. Informed consent was obtained from all subjects and/or their legal guardian(s). Reporting was done following the Consolidated Standards of Reporting Trials (CONSORT guidelines) (Fig. 1)^27^.

### Procedures

A total of fifty-two participants each receiving a Hawley retainer with embedded TheraMon® microsensor (MC Technology GmbH, Hargelsberg, Austria) were randomly allocated to receive either (A) audiovisual instructions and electronic weekly reminder (Audiovisual group) or (B) verbal only instructions (control group). Permuted block randomization ensuring 1:1 allocation ratio (block size = 4) was performed by independent personnel using a computer-generated number list. Allocation was concealed by using sequentially sealed opaque envelopes.

**Figure 2:**
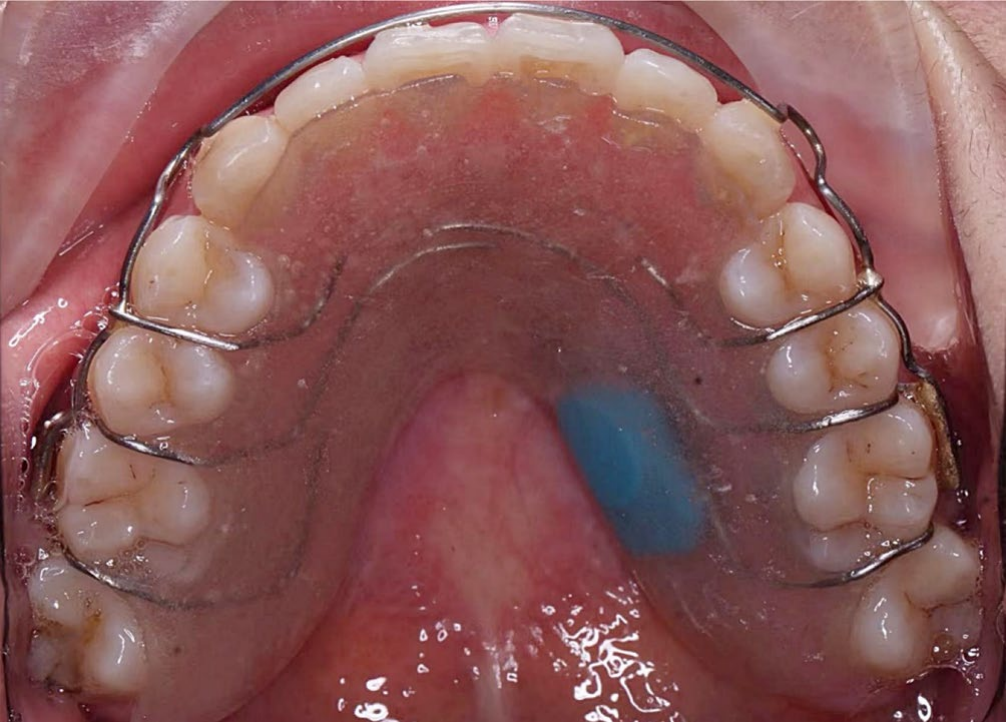
Microsensor embedded in upper removable Hawley retainer.

In debonding visit, all participants received standardized oral hygiene instructions with scaling and polishing procedures. Participants were instructed to temporarily wear a 1 mm thickness maxillary and mandibular vacuum-formed retainers (ACE®, DENTSPLY) for 3–4 days for 22 h each day until the Hawley retainer was manufactured. Standardized laboratory steps were undertaken by one experienced technician. Microsensors were embedded totally in the maxillary posterior palatal region of the acrylic covering (Palapress®; Kulzer GmbH, Mitsui Chemicals, Hanau, Germany) (Fig. 2). Complete coverage of the microsensors with acrylic ensures prevention from corrosion, and this region has shown better detection of intra-oral temperature changes^28^. A standardized coverage of 1 mm of acrylic was performed, to ensure optimum accuracy and precision of recording^29^.

**Figure 3:**
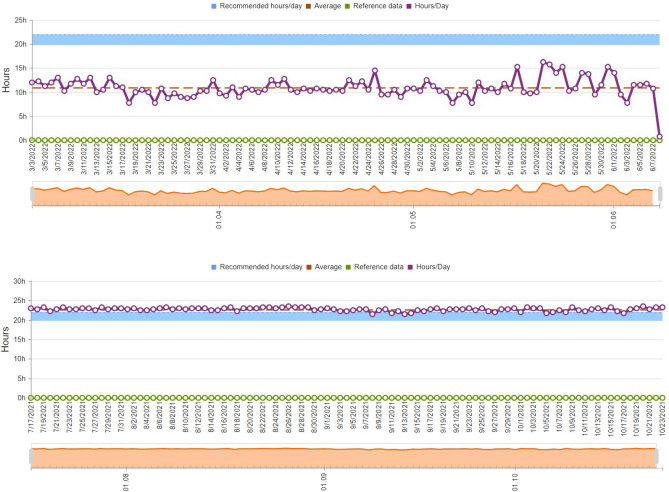
Daily wear times (in hours) over the 3-month period, upper graph shows sub-optimal compliance with 10 h of wear, lower graph shows a compliant patient with 23 h of daily wear.

At recall appointment (T0), participants in both groups were instructed to wear the Hawley retainer on a full-time basis for 22 daily hours for 6 months, with two follow-up visits after 3 months (T1), and 6 months from the start of retention (T2). Hawley retainer microsensor was activated at the same visit, by using a pen reader through an onboard antenna to the laptop, with attached TheraMon® client software (version 1.3.0.4, MC Technology GmbH, Hargelsberg, Austria). The microsensor was adjusted to monitor compliance wear within temperature range from 33.5 to 38.5 °C (attributable to variations in intra-oral temperature) every 15 minutes^30^. The microsensor uses 16 kilobytes of Electrically Erasable Programmable Read-Only Memory (EEPROM), which stores data for up to 100 days and can be reprogrammed multiple times for a maximum of 15 months. Thus, data was transferred after 3 months to the software cloud and was displayed as graphical compliance wear time over the 3-month period (Fig. 3). Additional detailed temperature graphs were displayed in case of missing data and to precisely evaluate records.

Participants’ gingival and plaque indices were recorded and compared at T0 and T2. Maxillary and mandibular periodontal measures were measured on all surfaces of indexed teeth 16, 36, 12, 32, 24/25, 44/45 to be a representative of the full dentition^31,32^. The original scoring system was used from 0 (normal) to 3 (severe), and then the mean value was measured for every patient. One examiner (K.W) performed the periodontal measures on all participants, while, a second examiner (J.Z) assessed 15 randomly selected patients to establish reliability of measurements and measurement error.

Participants completed an additional questionnaire at T2; the questionnaire assessed participants’ experiences and satisfaction with the Hawley retainer. Questionnaires were based on perspectives of the NHS, and patients’ subjective experiences^9^ which depend on questions that reflect patient satisfaction with used retention method and clinical outcomes. The primary version was piloted on 8 patients who were not involved in the original trial and were in their regular follow-up for retainer adjustment. Questionnaire papers were coded and assessed by one of the investigators. Participants were instructed to directly contact the hospital in case of any emergency, lost retainer, or feeling uncomfortable (sharp edge, ulcer, etc.). Dropouts were considered if a participant withdrew from the trial, failed to attend, or damaged the microsensor. A new Hawley retainer without a microsensor was manufactured instead. After the end of 6 months, patients were instructed to continue on part-time wear.

### Intervention

For the audiovisual group, participants watched four short videos upon delivery of the retainer (“Hold that smile—Why are retainers so important?’’, “Retainers keep your teeth from becoming crooked again”, and two additional illustrative videos on how to use and take care of the retainer)^21^. Videos were dubbed over with Chinese translation from the original English script, to involve full visual and auditory engagement. Thirty-six native Chinese speakers then verified the transcribed videos. Any recommended changes were applied to ensure reproducibility and accuracy in the main study. Additionally, a scheduled motivational reminder was sent weekly to the participants’ private WeChat mobile application. The reminders encouraged adherence to the prescribed daily wear, besides, emphasizing the importance of maintaining proper oral hygiene, and adherence to the scheduled appointments. For the control group, verbal and written instructions were only given to the participants. Both groups were aware of the presence of microsensors, and that they were being monitored.

The primary outcome was to assess the impact of the intervention on the adherence to the wear time instructions of the Hawley retainer in comparison to the control group, on a full-time basis (22 h) over a period of 6 months. The secondary outcomes were: (1) assessment and comparison of gingival and periodontal health after 6 months between the two groups, and (2) patients’ experiences and satisfaction with retainer wear.

### Sample size calculation

Sample size calculation was performed using G*power software (version 3.9.6.2 for Mac OS). A total sample size of 42 patients was sufficient to detect an effect size of 0.25 at a power of 0.95 (95%) and a partial eta square of 0.06 at a significance level of 0.05. To account for dropouts, the sample size was increased by 20% to 52 participants^33,34^.

### Statistical analysis

Statistical tests were performed by SPSS software 22.0 (Statistical Package for Social Science, Armonk, NY: IBM Corp) with an alpha level of 0.05 (significance set at *p* < 0.05). Descriptive statistics were calculated in the form of Mean ± Standard deviation (SD).

Inter-observer reliability was assessed using interclass correlation coefficient (ICC) between investigators and to assess measurement error.

For statistical analysis of outcomes, within-group outcomes were analyzed using paired sample *t* test to compare within-group ratings between T1 and T2 time points. Between-group outcomes were compared using independent sample *t* tests at each time point separately. The questionnaire results were analyzed using Chi-squared test for frequencies.

## Results

Fifty-two participants (mean age 26.1; range 16 to 35 years) were randomly assigned to either the audiovisual (26 participants) or control group (26 participants). Females represented almost two-thirds of the sample (69.2%) in comparison to males (30.8%). All participants were from the Chinese Han ethnicity. Three participants dropped out from the audiovisual group, and five from the control group (Fig. 1). The overall mean duration of wear in days was 99.2 (± 20.3 days) from T0 to T1 and 103.9 (± 24.1 days) from T1 to T2. The majority of participants were classified in the pre-treatment records as severe or handicapping on the Dental aesthetic index score. Accordingly, the majority of participants were treated with extraction-based treatment (Table 1).

**Table 1:** Baseline characteristics.

	Control group (26)	Audiovisual Group (26)	Total (52)
*Age in years*			
Mean ± SD	27.1 ± 1.3	25.0 ± 1.4	26.1 ± 1.0
*Gender (n)*			
Female	18	18	36 (69.2%)
Male	8	8	16 (30.8%)
*Ethnicity (n)*			
Han Chinese	26	26	52 (100%)
*Marital status*			
Married	12	10	22 (42.3%)
Single	14	16	s30 (57.7%)
*Highest education level*			
Junior High school	5	8	13 (25%)
Bachelor	18	16	34 (65.4%)
Masters	1	1	2 (3.8%)
PhD	2	1	3 (5.8%)
*The severity of malocclusion (Dental Aesthetic Index scores)*			
Mild (< 26)	0	0	0 (0%)
Moderate (26–30)	1	4	5 (9.6%)
Severe (31–35)	12	10	22 (42.3%)
Handicapping (> 35)	13	12	25 (48.1%)
*Extraction versus non-extraction*			
Extraction-based treatment	19	18	37 (71.2%)
Non-Extraction treatment	7	8	15 (28.8%)
*Appliances used before retention*			
Fixed appliances alone	18	18	36 (69.2%)
Fixed appliances plus miniscrews	8	7	15 (28.9%)
Fixed treatment/functional appliance	0	1	1 (1.9%)
Fixed appliance/expansion	0	0	0 (0%)

### Compliance with retainer wear

**Figure 4:**
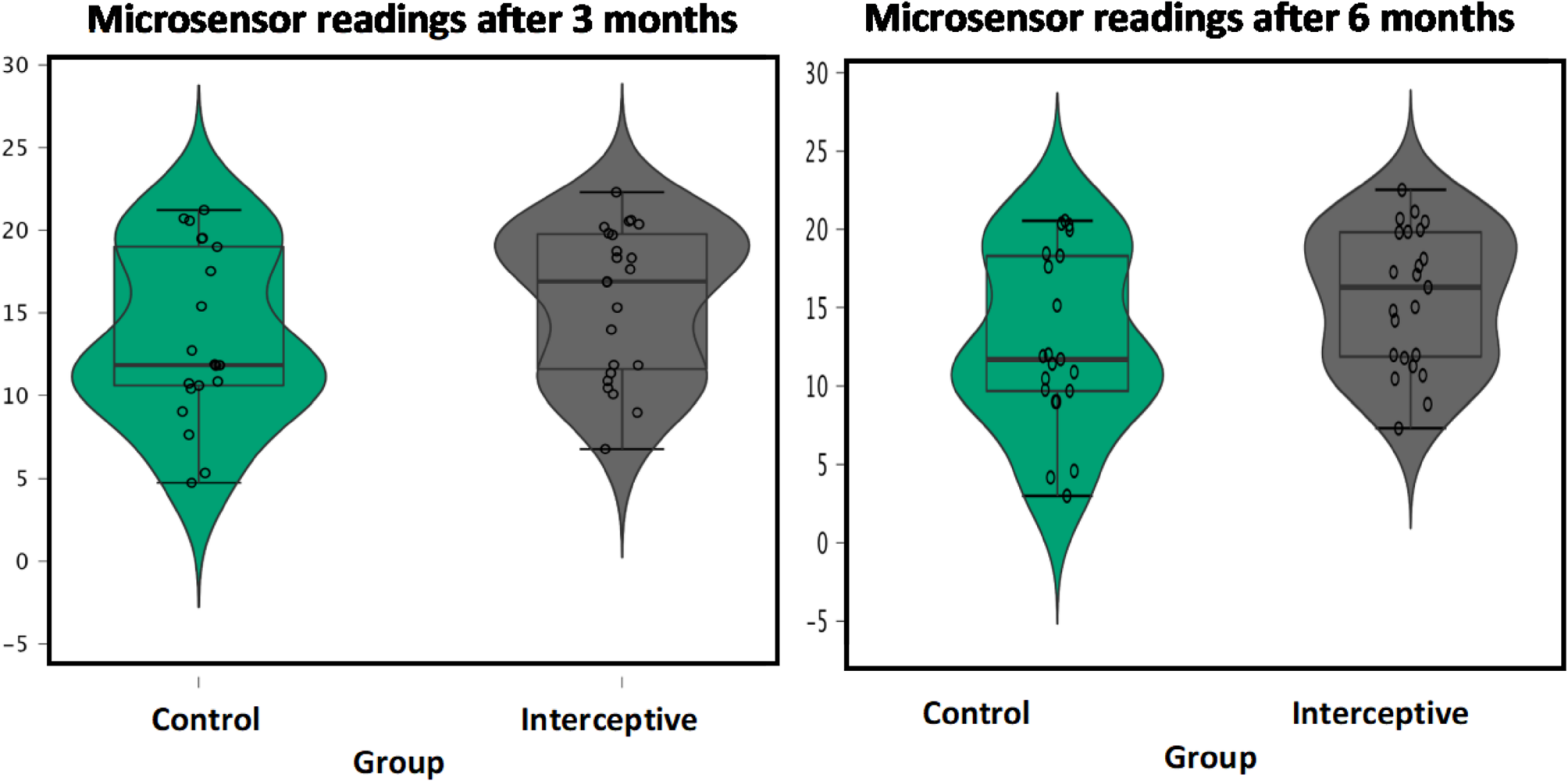
Boxplot presenting the microsensor readings after 3, and 6 months.

Overall, the mean objectively measured wear time in hours per day (h/d) after 3 months was 14.9 (± 4.9 h), and 14.3 (± 5.4 h) after 6 months. In terms of the mean duration of objective wear time between groups after 3 months (T1), the results were higher in the audiovisual group with (16.0 h) in comparison with the control group (13.7 h). At T2, the mean wear decreased for both groups with (15.6 h) for the audiovisual group, and (12.8 h) for the control group (Fig. 4). The microsensor readings were recorded as mean and standard deviation (SD), and are presented in Table 2.

(A) Intra-group compliance over time:

**Table 2:** Microsensor readings (in hours) recorded as mean and standard deviation.

Group	T1				T2				Paired t-test *p* value
	Mean		SD	95% CI	Mean		SD	95% CI	
Control	13.7	ab	5.1	11.4–16.1	12.8	c	5.5	10.3–15.3	0.001*
Audiovisual	16.0	a	4.5	14.0–18.0	15.6	a	4.4	13.7–17.5	0.170
T-test *p* value	0.065				0.033*				

*Significant at *p* < 0.05; T1, 3 months; T2, 6 months.

^a,b^Means followed by different letters vertically or horizontally are significantly different according to Duncan’s Multiple Range Test (DMRTs).

In the control group, the mean daily wear time significantly decreased from T1 (13.7 ± 1.0 h) to T2 (12.8 ± 1.1 h) (*p* = 0.001). However, in the audiovisual group, a non-significant trend was noticed from T1 (16.0 ± 0.9 h) to T2 (15.6 ± 0.9 h) as shown by the paired sample t-test (*p* = 0.170).

(B) Inter-group compliance over time:

After 3 months, a difference in the mean objective wear time (h/d) was noted with 13.7 ± 1.0 in the control and 16.0 ± 0.9 in the audiovisual group, however, a non-significant difference was observed between groups (*p* = 0.065). Nonetheless, objectively measured compliance in the audiovisual group (15.6 ± 0.9 h) was significantly higher than the control group (12.8 ± 1.1 h) at T2 (*p* = 0.033).

### Gingival and periodontal health

Agreement between both examiners in periodontal measurements was excellent (ICC: 0.92).

(A) Gingival index

Intra-group results have shown significant improvement in the gingival index scores after 6 months with (*p* = 0.042) and (*p* = 0.014) for the control and audiovisual groups, respectively. However, for inter-group results, there was a non-significant difference at T0 (*p* = 0.233), and at T2 (*p* = 0.165) (Table 3).

(B) Plaque index

**Table 3:** Gingival and plaque scores recorded as mean and standard deviation.

Group	T0			T2			T2–T0				Paired t-test *p* value
	Mean		SD	Mean		SD	Mean	SD	% change	95% CI	
*(A) Gingival index*											
Control	0.17	a	0.36	0.04	b	0.08	− 0.13	0.08	− 79.20	− 0.29 to 0.02	0.042*
Audiovisual	0.33	a	0.54	0.12	b	0.27	− 0.21	0.27	− 63.71	− 0.38 to − 0.05	0.014*
T-test *p *value	0.233			0.165				0.494			
*(B) Plaque index*											
Control	0.16	ab	0.19	0.14	c	0.20	− 0.02	0.01	− 14.44	− 0.10 to 0.05	0.313
Audiovisual	0.16	a	0.27	0.04	b	0.11	− 0.12	0.16	− 77.22	− 0.23 to − 0.08	0.007*
T-test *p *value	0.629			0.173				0.142			

*Significant at *p* < 0.05; T0; start of treatment, T2; after 6 months.

^a,b^Means followed by different letters vertically or horizontally are significantly different according to Duncan’s Multiple Range Test (DMRTs).

Intra-group results have shown a non-significant improvement in the control group after 6 months (*p* = 0.313), however, significant improvement in the plaque index scores was noted in the audiovisual group at T2 (*p* = 0.007). For the inter-group results, no statistical significance was observed between both groups at any given time point, with (*p* = 0.629) and (*p* = 0.173) for T0 and T2, respectively (Table 3).

### Patient experiences

Forty-four participants completed the questionnaire at the end of the study, twenty-one from the control group, and twenty-three from the audiovisual group (Table 4). Participants in both groups underestimated their wearing time by (1.4 and 1.7 h) in the audiovisual and control groups respectively. Majority of participants used to wear and remove the retainers in the first 4 days (47.8% and 52.4%). In the audiovisual group, the majority of participants adapted to talking with the retainer in the first 4 days (47.8%), however, in the control group, it took 5–8 days to get used to it (42.9%). Majority of participants in both groups have shown the ability to take care of the retainer (85.7% and 82.6%), or cleaning it (81.0% and 78.3%). In the audiovisual group, none of the participants reported feeling stressed about the microsensor. In the control group, few participants had some fear of being monitored (4.8%). For patient satisfaction with the delivering way of instructions; all participants were satisfied with the used methods. However, in the audiovisual group, participants were very satisfied with retainer instructions (47.8%), in comparison to the control group (14.3%).

**Table 4:** Results of patient experience questionnaire.

Variable	Description					Chi-square *p* value
*How many hours (on average) per day have you worn the retainer?*						
Control	1.7 h					0.157
Audiovisual	1.4 h					
*How many days does it take to get used to wearing and removing of the retainer?*						
Control	1–4 d 11 (52.38%)		5–8 d 8 (38.10%)		9 ≤ d 2 (9.52%)	0.014*
Audiovisual	1–4 d 11 (47.83%)		5–8 d 8 (34.78%)		9 ≤ d 4 (17.39%)	
*How many days does it take to easily talk with the retainer?*						
Control	1–4 d 6 (28.57%)		5–8 d 9 (42.86%)		9 ≤ d 6 (28.57%)	0.045*
Audiovisual	1–4 d 11 (47.83%)		5–8 d 7 (30.43%)		9 ≤ d 5 (21.74%)	
*Was it easy to take care of your retainer at school/university/work?*	Yes			No		
Control	18 (85.7%)			3 (14.3%)		0.046*
Audiovisual	19 (82.6%)			4 (17.4%)		
*Was it easy to keep your retainer clean?*	Yes			No		
Control	17 (81.0%)			4 (19.0%)		0.0455*
Audiovisual	18 (78.3%)			5 (21.7%)		
Have you felt stressed about having a microsensor in your retainer?	Yes			No		
Control	1 (4.8%)			20 (95.2%)		0.317
Audiovisual	0 (0%)			23 (100%)		
*How do you evaluate the clinician instructions about the importance of the retainer?*	Very dissatisfied	Dissatisfied	Neither satisfied nor dissatisfied	Satisfied	Very satisfied	
Control	–	–	–	18 (85.71%)	3 (14.29%)	0.045*
Audiovisual	–	–	–	13 (52.17%)	11 (47.83%)	

*Significant at *p* < 0.05; d, days; h, hour.

## Discussion

Objective wear time of both groups demonstrated sub-optimal compliance with instructed wear time. The utilization of audiovisual instructions has shown a statistically significant positive effect on improving adherence with the recommended wear time in comparison to delivering verbal instructions only after 6 months. Nonetheless, periodontal outcomes did not differ between both groups. Participants’ experiences were similar in both groups, except for satisfaction with the way of delivering instructions, being favorably perceived by patients receiving the additional audiovisual instructions and weekly reminders.

The assigned audiovisual instructional videos were chosen to promote the need to wear the retainer and were supplemented with weekly reminder messages on the WeChat application. A significant positive effect of the used protocol on compliance with retainer wear after six months was observed. This goes in line with results from previous literature, which did show an improvement in information retention^18,22^. However, the overall compliance was still suboptimal and this could be due to the nature of Hawley retainer that affects appearance, speech, or other multi-faceted factors that affect compliance. In previous literature, the applicability of having significant improvement in gained knowledge using digitized methods does not affect compliance levels and this may relate to the fact that these studies were only done over short term periods^26^. Similar results were noticed in our study, by having a significant effect over extended follow up time. This could demonstrate that reminders were more effective on the long term in comparison to shorter periods of observance^13,25^. Moreover patients could have been affected by the Hawthorne effect in two different ways; the first way is through their observance of the clinicians’ constant weekly reminder messages, while the other is through their awareness of the presence of the microsensors.

The delivered audiovisual videos additionally focused on oral health promotion. Results have generally shown a non-significant difference between the groups after 6 months. Both groups had a significant improvement in oral health readings over time, except for the plaque scores in the control group. This is consistent with previous literature which has shown that significant deterioration in oral health occurred at the end of orthodontic treatment, which gradually improved after debonding^35^. Our results align with previous literature, by not having an additional value from the utilization of digitized motivational methods to improve oral health^26^. This is contrary to the results that were observed during orthodontic treatment^36^. These contradicting results could be due to the difference in the nature of oral health preferment in treatment and afterward. After treatment, oral health readings improved for all patients over time, as patients were able to clean their teeth properly with the removable appliances. However, during treatment, behavioral interventions could be beneficial to motivate patients on oral health maintenance in comparison to the retention phase.

Participants’ experiences were more or less similar in both groups, except for satisfaction with the way of delivering instructions, being favorably perceived in the audiovisual group as recorded via the questionnaire. The administered questionnaire was developed depending on the perspectives of the National Health Service, which depend on questions that reflect patient satisfaction with the Hawley retainer, and the impact of clinical methods of communication and their results. Questionnaires were given to each participant after 6 months of observation to reflect their perspectives after a period under retention. Results demonstrate the benefit of the multimodal approach in delivering clear information in comparison to the usual methods^22^. Participants underestimated their wearing time, which is contrary to previous literature that has shown a general overestimation of wearing hours^37^. This may be explained by the fact that participants knew that they were being monitored. Interestingly, few participants expressed their stress of being monitored in the control group, which may relate to the importance of the way of delivering information. Moreover, the majority of participants reported being comfortable with the retainers in the first week and feeling less stressed.

The choice of the retainer type and retention protocol is still debatable in literature. In some practices, Hawley retainers are still preferred for their known advantages in occlusal settling, and their capacity to have incorporated modifications for simple teeth movements during retention. However, they are often associated with temporary speech changes and less desirable aesthetics due to the metal labial bow. In this trial, we aimed to reflect what aligns with traditionally known clinical practices^38,39^. Moreover, microsensor failures are higher in vacuum-formed retainers in comparison to Hawley retainers, which would further complicate the long term follow up and compliance monitoring in patients receiving vacuum-formed retention^29^.

Effective communication and compliance are integral parts of successful treatment. Understanding effective ways of delivering information helps in the establishment of an effective rapport with clinicians. The information provision process must ensure that the patient receives, understands, and retains the information over long periods. The use of multimodal approaches by mixing the auditory and visual senses helps to retain information for a longer duration^19^. On the contrary, the use of single method of communication (verbal, written, or visual), has shown a non-significant effect on the retention of information on patients and their families^18^. The multi-faceted nature of information retention depends on the positive expressive reaction that results in relaxing behavior and full capacity of concentration^17^. This aligns with our results that show significant improvements in participants’ knowledge by using the audiovisual information through instructional videos, and additionally through mobile application reminders.

Digitized methods of communication and monitoring have recently accelerated the transformation toward the use of digital information and communication technologies^40^. This could help in monitoring and analyzing large data about patients’ behavior and factors affecting compliance^41^. These digitized methods helped to cope with upcoming challenges in the orthodontic field in recent years^42^. The development of innovative methods aids in exploring alternative ways for modeling patient behavior. For instance, the utilization of new means to retain information by providing audiovisual illustration will enhance information retention^22^. Furthermore, the use of sensor-based technologies has paved the way for future modifications that could integrate interactive thermal and wearable sensors, depending on personalized monitored data, which would guide the process more precisely.

### Limitations

Microsensors have shown substantial accuracy in *in-vitro* studies, while clinically, they under record actual time in the palatal surface by 1.2 h^43,44^. All patients were informed about the presence of recording microsensors which may influence their wear behavior^37^. Some technical problems occurred with one participant when reading the microsensor after 3 months, leading to loss of data. The follow up is only a reflection of compliance on a full-time basis over a 6-month period. Further, high-quality research is needed to reflect longer durations and compliance with part-time wear. A Hawley retainer was used, which might be less preferable by the patients, thus, this can discourage overall participant compliance in comparison to other options. Patients with sub-optimal compliance with retainer wear still managed to fit their retainers by the end of the observation period. Their realization that they were wearing their retainers for the right time (i.e., by observing no-to-minimal changes in their dentition) despite the issued instructions could be one of the reasons behind their decision. These observations are further supported by recent evidence suggesting that reduced wear times of removable appliances were sufficient to achieve good outcomes^45^.

## Conclusions

Audiovisual instructions combined with weekly reminder messages seem to have a significant effect on improving compliance with the removable Hawley retainer wear. Nonetheless, periodontal health did not change significantly when audiovisual instructions were implemented. Participants’ experiences were similar, except for satisfaction with the way of delivering instructions, being favorably reported in the audiovisual group.

## Data Availability

Datasets are available with the corresponding author upon reasonable request.
